# Is Africa ready for mobile colistin resistance threat?

**DOI:** 10.1080/20008686.2021.1962781

**Published:** 2021-08-04

**Authors:** Madubuike Umunna Anyanwu, Ishmael Festus Jaja, James Wabwire Oguttu, Chinwe Juliana Jaja, Kennedy Foinkfu Chah, Vincent Shodeinde Shoyinka

**Affiliations:** a Department of Veterinary Pathology and Microbiology, University of Nigeria, Nsukka, Nigeria; b Department of Livestock and Pasture Science, University of Fort Hare, Alice, South Africa; c Risk and Vulnerability Science Centre, University of Fort Hare, Alice, South Africa; d Department of Agriculture and Animal Health, University of South Africa, Roodepoort Johannesburg, South Africa

**Keywords:** Africa, *mcr*, antibiotic resistance, colistin, emerging threat

## Abstract

Antimicrobial resistance is a growing public health problem and a threat to effective treatment and prevention of an array of infections caused by bacteria. Africa is already faced with many socio-economic and health crises. Many countries in Africa can seldom boast of a standardized health care facility comparable to those in developed countries. Yet, the non-therapeutic use of COL has been banned in developed countries. However, in Africa, except for South Africa, COL is an over-the-counter (OTC) medication sold and dispensed by non-professionals/without a veterinarian’s supervision. The ban of non-therapeutic COL in developed countries has proven to reduce the development of mobile colistin resistance (MCR) in humans and animals. The unregulated use of COL has been proven to select pathogenic and commensal bacteria resistance. A transmissible plasmid-mediated colistin determinant, mobile COL resistance (*mcr*) gene, which is rapidly transferred/acquired horizontally or laterally intra/inter-species/genera, has been reported. A highly promiscuous mobile genetic element like plasmids containing transposons, insertion sequences, and integrons aid the carriage/rapid transfer and acquisition of these *mcr* genes. Hence, we highlight the danger posed by escalating colistin (COL) resistance in the continent and the impetus to halt the indiscriminate and non-therapeutic use of COL to protect public health.

## Introduction

For many decades, antibiotic has revolutionized healthcare globally, bringing respite to clinicians and veterinarians, especially regarding the treatment of stubborn infections. But the increasing problem of antimicrobial resistance threatens to reverse all the benefits of antibiotics in modern medicine. The damaging impact of antimicrobial resistance (AMR) is already manifesting around the globe. For instance, over 50,000 lives are lost each year across Europe and the US alone, and hundreds of thousands more are dying in other parts of the world[1]. The global estimate of deaths due to AMR stands at 700,000, and it is projected to reach 10 million by the year 2050[1]. In Africa, deaths attributable to AMR every year by 2050 are estimated to reach 4,150,000[1]. The projected figure could even be higher in low- and middle-income countries and specifically in Africa, where there is a lack or absence of health infrastructure, poor hospital seeking habit, poor record-keeping, and generally weak or poor governance and regulatory framework. Hence it is time to sound the alarm on the trend and spread of colistin resistance in Africa and the need for every country in the continent to enhance its antibiotics stewardship programs.

## Mechanisms of colistin resistance

Colistin (COL) is a polymyxin antibiotic used as a last-line agent for treating deadly infections in humans and animals. COL was discovered in 1947 and isolated in 1950 from the soil bacterium *Paenibacillus* (*Bacillus*) *polymyxa* subspecies *colistinus* [2]. But its use in human medicine was largely abandoned in the 1970s due to its toxic effects on the kidney and nervous system. However, it continued to be used in the livestock sector in most parts of the world, including inAfrica [3]. However, in the early 2000s, the use of COL in humans was reintroduced due to the rapid rise in deadly infections caused by multi- and extensively-drug resistant organisms [4].

There was a low interest in the COL resistance concept because it was thought that the only mechanism for COL resistance is by mutation in chromosomal genes such as pmrAB, phoPQ, mgrB, and crrAB which are only vertically transferred to bacterial progenies (clones), and thus, by its very nature rare and self-limiting. This perception changed in late 2015 following the discovering of a transmissible plasmid-mediated colistin determinant, mobile COL resistance (*mcr*-1) gene (in *Escherichia coli* isolates from humans/meats in China), which is rapidly transferred/acquired horizontally/laterally intra/inter species/genera [5]. After the discovery in China, subsequent detection of this plasmid-mediated mechanism was quickly reported worldwide [6].However, retrospective studies have shown that *mcr* gene has been in existence since the 1980s. This coinciding with when COL use in livestock began in China but remained undetected [7]. The rapid transfer and acquisition of *mcr* gene is because they are carried by highly promiscuous mobile genetic elements like plasmids containing transposons, insertion sequences, and integrons. Thus, with mobile colistin resistance (MCR) fast-spreading globally, this poses a considerable threat to antimicrobial therapy, especially in Africa where access to other good quality effective last-line antibiotics is limited.

## Distribution of colistin resistance in Africa and its implications

**Figure 1.**
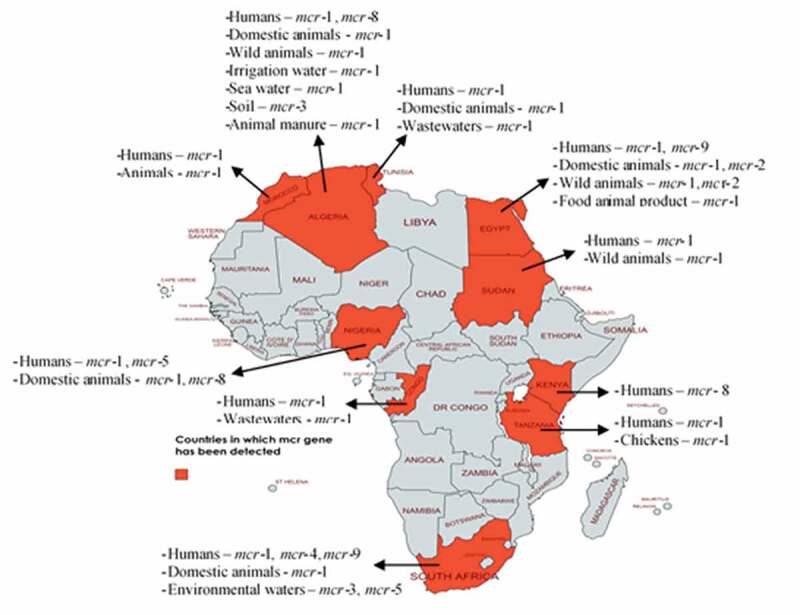
Mobile colistin resistance mechanisms in diverse ecosystems in Africa. This map was created using an online service (https://mapchart.net/)

Available evidence shows that these genes have been detected in isolates from all regions of Africa (Figure 1). There are ten *mcr* gene types (*mcr*-1 to *mcr*-10), and of these, include *mcr*-1 the predominant one, other genes such as *mcr*-2, *mcr*-3, *mcr*-4, *mcr*-5, *mcr*-8, and *mcr*-9. All these have been detected in Africa (Table 1). These *mcr* genes evolved and are disseminated by various plasmids such as IncHI2, IncP, IncI, IncX4, IncN, IncR, transposons/insertion sequences (especially IS*Apl1*), and class 1 integrons (Table 1). The genes are trafficked by a diversity of bacteria, including predominantly *Escherichia coli, Salmonella, Klebsiella, Citrobacter, Enterobacter, Pseudomonas, Acinetobacter*, and *Alcaligenes faecalis* isolated from humans, animals, food of animal origin, and environment [8–11]. This is not surprising because while COL use in humans is uncommon in Africa, its use in livestock is largely unregulated [12].

**Table 1.** Ecological niches in which mobile colistin resistance gene has been detected in Africa

Region	Country	mobile colistin resistance gene	Sources	*mcr*-carrying organisms	Plasmids/other genetic elements associated with *mcr*	*mcr*-positive organisms contain gene(s) coding other last-resort antibiotics	References
Northern	Tunisia	*mcr*-1	Humans, food animals (chickens, camels and cattle), and food animal products (chicken meat, beef and cattle milk) and wastewaters	*Escherichia* (*E*.) *coli*	IncHI2, IncP, IncI2, IncI1, IncFIB	Yes	[16,25,29,35–39]
	Algeria	*mcr*-1, *mcr*-3 and *mcr*-8	Humans, chickens, wild monkey, agricultural soil, horse manure, irrigation and sea water	*E. coli* and *Klebsiella*	IncHI2, IncP and transposon IS*Apl1*	Yes	[18,20,22,40–46]
	Egypt	*mcr*-1, *mcr*-2 and *mcr*-9	Humans, food animals (cattle and chickens), wild migratory and resident birds, food animal products (beef, chicken meat and cheese), and surface water	*E. coli, Acinetobacter baumannii, Pseudomonas* and *Enterobacter*	IncHI2, IncI1, IncI, IncFIB, IncX4, IS*Apl1* and class 1 integron *Int*1	Yes	[9,15,17,47–58]
	Morocco	*mcr*-1	Humans and chickens	*E. coli* and *Klebsiella*	-	Yes	[31,54]
Southern	South Africa	*mcr*-1, *mcr*-3, *mcr*-4, *mcr*-5 and *mcr*-9	Humans, food animals (chickens, cattle and pigs), sewage, rivers and storm waters	*E. coli, Enterobacter Klebsiella* and *Acinetobacter*	IncN, IncHI, IncX4, IncI2, IS*Apl1*and IS*3*	Yes	[28,59–66]
Central	Congo Brazaville	*mcr*-1	Humans and wastewaters	*Pseudomonas aeruginosa*	-	-	[11]
Eastern	Tanzania	*mcr*-1	Humans and chickens	*E. coli*	IncX4	Yes	[34]
	Sudan	*mcr*-1	Humans and wild fennec foxes	*E. coli, Pseudomonas* and *Klebsiella*	-	Yes	[10,30,67]
	Kenya	*mcr-8*	Humans	*Klebsiella pneumoniae*	IncHIB and IncR	Yes	[68]
Western	Nigeria	*mcr*-1, *mcr*-5 and *mcr*-8	Humans, food animals (chickens and pigs)	*E. coli, Enterobacter, Klebsiella, Citrobacter* and *Alcaligenes*	IncX4	Yes	[8,69]

Moreover, it has been established that antibiotics induce selective pressure in bacteria prompting them to acquire resistance genes. Thus, there is a high likelihood that in Africa, MCR originated in the veterinary sector from where they disseminated to other ecosystems. Like other drugs in most African countries except for South Africa, COL is an over-the-counter (OTC) medication sold and dispensed by non-professionals/without a veterinarian’s supervision. Majority of Africans, including livestock farmers, have poor knowledge about antibiotics and antibiotic resistance. Hence they patronize quacks (non-professionals) and engage in self-medication thereby increasing the problem of resistance. Besides, it is also possible that some of the COL that is sold on the market in these nations are counterfeit or of low quality. Moreover, many antimicrobials used in human and animal medicine in Africa are mostly unregulated [13]. Yet, these non-polymyxins also facilitate COL resistance [14]. Thus, MCR tends to occur in conjunction with resistance against many other antimicrobial agents, making organisms multi to pandrug resistant. This further leads to limited options for therapy. This situation is exacerbated when an incriminated organism contains *mcr* and gene coding resistance against other last-resort antimicrobials such as fluoroquinolones, extended-spectrum cephalosporins, fosfomycin, and tigecycline. Unfortunately, such organisms, also referred to as ‘superbugs,’ have already been isolated from humans [15], animals [16], and food of animal origin [17] in Africa.

Of noteworthy is that in Africa (Figure 1), *mcr* genes (*mcr*-1 and *mcr*-2) have been detected in wildlife (terrestrial and aquatic) where antibiotics are not used [18,19], thus implying that MCR has disseminated to all ecological niches in Africa. Wildlife in Africa has also been associated with disseminating *mcr* genes into the environment (surface waters) and the human population [19]. The latter may happen through water used for cooking, laundry, bathing, drinking, and food processing. The genes in the organism from the surface waters could also come from other parts of the world. For example, *mcr*-1 has been detected in isolates from the sea in Africa [20].

Handling, coming in contact with, and consuming contaminated food of animal origin and fomites, is also a putative source for acquiring *mcr* genes. Unfortunately, unhygienic animal slaughter techniques are employed in the majority of the slaughterhouses in Africa, and worse still, in Africans, especially those in rural areas, humans live in very close contact with their livestock [21].

## Possible modes of colistin gene transfer in Africa

Improper disposal of livestock/slaughterhouse wastes into the environment and their use as organic fertilizer in farmlands and aquaculture can spread *mcr* genes into the environment. For example, *mcr*-1 has been detected in irrigation and sea waters in Africa [20,22]. Through the environment, *mcr* genes are reincorporated into the food chain through contaminated irrigation water, soil, crops, and wildlife [23]. Furthermore, poor personal hygiene, poor waste management, and poor environmental sanitation are prevalent in most African countries and households. This facilitates the spread of *mcr* genes (Figure 1) within the human population and other ecosystems due to practices like open air defecation that are still common in Africa.

## How prepared is Africa?

MCR has an immense One Health ramification in Africa and therefore warrants urgent intervention. With the world gradually turning into ‘one village’ due to ease of travel between countries and continents, the inability to urgently control the spread of MCR in Africa can potentially result in an outbreak of difficult-to-treat diseases in humans and animals worldwide, which could have catastrophic impacts. The seriousness of this is appreciated when one considers that some of the *mcr*-harbouring organisms from Africa, especially the *E. coli*, belong to the high-risk zoonotic pandemic extraintestinal pathogenic *E. coli* (ExPEC) clones [22,24,25]. Furthermore, globally, diseases associated with multidrug-/extensively drug-resistant organisms were recently reported to cause an estimated 700,000 human deaths annually [1]. The question that arises is whether Africa is ready to face a threat posed by MCR in the absence of effective antibiotics that can destroy superbugs.

Antimicrobial stewardship (AST) is a proven way to curb antimicrobial resistance. Education of human and animal health workers and the public on prudent/judicious use of antibiotics, conduct of antimicrobial sensitivity test before prescription of antibiotics, and infection prevention and control (IPC) practices like hand hygiene, forms the core of AST program. Unfortunately, Africa is far behind regarding establishing and practicing AST programs [26]. For example, the ban on the non-therapeutic COL has proven to reduce MCR development in humans and animals [27]. But this policy is not yet in view in Africa because weak national drug regulatory authorities characterize most countries in Africa except a few like South Africa, inefficient antibiotic policies, and erratic access to antibiotics [26]. Getting involved in globally-coordinated antimicrobial surveillance programs is needed to control the importation and exportation of *mcr* genes into/from Africa. Assessing for *mcr* genes among travelers returning to Africa and quarantine and testing animals imported into the continent is vital to control MCR’s spread. The justification for evaluating and testing both travelers and imported animals is not far-fetched. Some *mcr*-harboring isolates from sick individuals in South Africa were reported to be unrelated to any African strain [28]. The later suggests, that medical tourism could be promoting acquisition of MCR.

Furthermore, through the food animal trade route, *mcr*-1 was imported from France into Tunisia [29]. On the other hand, wild animals captured in Sudan were vectors for exporting *mcr*-1 to China [30]. Human movement also plays an important role in the spread of *mcr*-1. For example, Moroccans and Algerians who traveled to Mecca for Hajj pilgrimage imported *mcr*-1 on returning to their home countries [31]. A citizen of Algeria transported *mcr*-1 to France [32]. An American and English person who visited Kenya and Egypt, respectively, carried *mcr*-1 back home [32,33], and *mcr*-1 was detected in isolates from hotel workers in Tanzania that often make contact with foreign tourists [34].

Since as *mcr* gene has been isolated from sea waters in Africa [20], it can be carried by water current to other parts of the world. Therefore, efforts need to be stepped up in Africa to control the spread of MCR to preserve the efficacy of COL.

## Conclusion

In conclusion, mobile COL resistance in Africa is a substantial public health issue with the potential to spread to other continents. However, the continent is not yet ready for this threat. This is a health catastrophe in the making if aggressive efforts to control the development and spread of MCR in Africa are not implemented immediately.
